# Supplementary material for: Balrog: A universal protein model for prokaryotic gene prediction
Source: PLoS Comput Biol. 2021 Feb 26;17(2):e1008727. doi: 10.1371/journal.pcbi.1008727 (PMC7946324; doi:10.1371/journal.pcbi.1008727)
Supplement: Attachment — Submitted filename: Balrog_review_response_letter_.docx [file pcbi.1008727.s004.docx]

**Author response to reviewer comments on “Balrog: A universal protein model for prokaryotic gene prediction”**

Markus J. Sommer & Steven L. Salzberg

We would like to thank both reviewers for their insightful comments and questions. Considering the issues raised, we have significantly revised the manuscript and released a new, portable version of Balrog, completely rewritten in C++ for standard CPU architectures. We hope this change will provide more stability to users in addition to substantial speed increases. The C++ version is available with “conda install balrog --channel bioconda” and precompiled binaries will be made available for Linux/macOS systems in the coming weeks.

The C++ version is currently hosted at <https://github.com/Markusjsommer/BalrogCPP>

Please find our point-by-point responses to all comments below. Our responses bolded to distinguish them from the reviewers’ comments:

Reviewer #1:

I am glad to see some new tool developed using deep learning to predict proteins from prokaryote genomes and this sounds a nice tool to improve the accuracy and easily used without training for specific taxonomic units like other tools, e.g. prodigal and prokka. I would like to test it by myself, but failed in using the web server provided by the authors, even failed in uploading my sequences. I suppose the model file is large hindering distribution of the tool. Standalone version is much helpful.
The writing is good, then I still have other concerns.

**To address this concern, we have implemented and released a new, portable C++ version of Balrog, hosted by Bioconda and available with “conda install balrog –channel bioconda”. Moving from python to C++ and reorganizing how ORFs are scored has allowed us to achieve acceptable run times while removing the dependency on GPUs. Moving forward, we intend for the CPU C++ version to be the main supported release for Balrog. This will eliminate the reliance on Google’s servers, which we hope will enable a more stable user experience. Note that the Google Colab version still works as before.**

**As of 12-20-20, the first release of BalrogCPP does not yet include the MMseqs2 integration which helps reduce false positives in the python version. Additionally, some users have already reported pytorch dependency issues with cpu instruction sets. This will be fixed in an upcoming release.**

1) As we know, prokaryote genome sequnces are largely biased in sequncing for some pathogens. Then the data set for taining is not balanced.

**We have added more information highlighting this issue and discussing our attempt to capture prokaryotic diversity while limiting the impact of highly overrepresented species:**

**“As a whole, currently available prokaryotic genomes are biased toward clinically relevant organisms. Many low-abundance environmental species may be absent from public databases, whereas organisms important to human disease may have full genomes for hundreds of closely related strains \cite{Almeida2019-xz}. We cannot fully account for the missing diversity within available sequence databases (indeed, millions of bacterial species probably remain unsequenced), but to limit the impact of highly-overrepresented species, we randomly selected only one genome for each bacterial and archaeal species within the Genome Taxonomy Database (GTDB, https://gtdb.ecogenomic.org) for the training set \cite{Parks2020-wz}.”**

2) for prokaryote genomes, the difference of gene numbers within the same species, that is, different populations/strains, is large because of HGT or other reasons resulting in quite difference if accessory genomes. Why the authors select proteins for training based on the rule of picking up genomes and then determine the proteins. It looks like that the authors need to select all prokaryote genomes with high qualtiy and then cuurate pangenome to cluster these proteins for traing your model. Another option is that the author could extract high quality of protein seuqnces of porkaryotes from known databases, e.g. uniprot.

**This point came up during initial discussions of how to train our model. Ultimately, we felt the most interpretable and fair comparison could be made using genome-level metrics. We have added a discussion of this approach to the text:**

**“An alternative approach to training a universal protein model could use protein clusters to capture diversity in protein sequences with less redundancy than our whole-genome approach. However, we wanted our final evaluation metric to be as fair as possible to all gene finders and reflective of a real-world situation where a newly sequenced prokaryote would likely contain many proteins from many different clusters.”**

**We would add (although this is not in the manuscript) that a test set using the protein clustering approach would necessarily exclude random protein clusters from training, then measure performance on these excluded test clusters. We felt that this would result in a relatively abstract way to test performance which was not as interpretable as training on proteins from whole genomes and testing on held-out genomes from distantly related organisms. Also, we would note that our training/testing regime mirrors the methods used to evaluate previously published bacterial gene finders.**

3）I am not sure the rule to make non-hypothetical genes only based on a description containing “hypothetical” or “putative”. This is really coarse.

**We agree this is imperfect, but “hypothetical protein” is based on a long standard naming convention set by RefSeq using stringent quantitative criteria. We have added more specific language to the text describing why proteins are labeled hypothetical:**

**“In standard annotation pipelines, proteins are labeled hypothetical if they have no significant match to known protein sequences and are not otherwise covered by a standard naming rule \cite{Haft2018-cp}.”**

4) two figures are too simple to express clearly what was done by authors.

**We have added Figure 3, a flowchart detailing all steps from genomic sequence input to gene prediction output.**

Reviewer #2: 

Major points:

1) It is unclear to what degree the training set is biased by the fact that many gene annotations in the training genomes are also produced by bioinformatic prediction tools. Since Glimmer3 and Prodigal have been the standard tools for gene prediction since 1998 and 2010, respectively, it is likely that most of the 'extra" genes annotated as hypothetical were actually predicted found by Glimmer3 or Prodigal. It is therefore not surprising at all that Glimmer3 and Prodigal would find more such 'extra' genes than a tool such as Balrog using a very different methodology.

**It is true nearly all current annotations have been made by Glimmer, Prodigal, or Genemark. This makes it nearly impossible to prove Balrog has a higher true positive rate, i.e. even if Balrog were to find more true proteins, these proteins would have been missed in the annotation made by the other programs and would not be labeled correctly.**

**However, we can show that Balrog finds the same number of true positives while predicting less proteins overall. This is what we have done, and it is essentially beating Glimmer and Prodigal at their own game, as they were responsible for the original annotations.**

**Reduction of hypothetical predictions was the metric used by both Glimmer and Prodigal (in previous publications) to demonstrate improvements between versions and programs. We feel it is a fair, if imperfect, way to show performance improvements on a large scale.**

**We have attempted to clarify our reasoning behind our metric in the text:**

**“In line with evaluation metrics used by other gene finders, if a program can find nearly all true positive genes while predicting fewer genes overall, it is reasonable to assume this is primarily due to a reduction in false positive predictions \cite{Delcher2007-cd, Hyatt2010-aw}. Thus, we would prefer a method that makes fewer overall predictions while retaining very high sensitivity to known genes.”**

The authors need to construct a benchmark that can correct for such biases or at least estimate them. One option could be to test on genomes that have been annotated using experimental data such as RNA-seq, CAGE-seq or the like.

**It is reasonable to think experimental evidence may help us compare gene finder performance, even if on a single genome, but we could not find evidence to support this. Unfortunately, experimental data such as RNA-seq and CAGE-seq, as valuable as they are, are not accurate enough to improve the annotation of prokaryotes. We are not aware of experimental techniques that definitively show that a gene is false positive, which is even harder to show.**

**RNA-seq experiments can be used to measure gene expression in bacteria. However, it’s much more difficult to use them to find genes, in part because of the operon structure of bacterial genomes. Genes are often expressed in polycistronic units, with many genes transcribed together. The sequences between those genes might have open reading frames even though they are not protein-coding, which could easily lead to many false positives. For different reasons, CAGE-seq (Cap Analysis Gene Expression) provides an experimental method to verify the 5' end of mRNA in eukaryotes, but it is ``not applicable to prokaryotes" \cite{Takahashi2012-ur}.**

2) It would be important to get more information on how much this very highly parameterized method can generalize beyond the genus. The benchmark should therefore be repeated with training sequences from which all genomes from the same family / order of any of the test genomes have been excluded.

**We have repeated the full gene model training process after removing all genomes sharing a family rather than genus with any organism in the test set. The results were almost exactly the same, with 98.12% sensitivity on average, versus 98.15% with the genus-level information was excluded. To keep Table 1 a reasonable size, we have added summarized results to the text:**

**“Similar results were observed when the gene model was trained on a set excluding organisms sharing a family, rather than a genus, with any organism in the test set. On average, the gene model achieved sensitivity of 98.12\% with family excluded vs. 98.15\% with genus excluded (2247 vs. 2248 genes) in bacteria and 97.50\% vs. 97.44\% (1662 vs. 1661) in archaea. The family-excluded model predicted on average 25 more extra genes than the genus-excluded model in bacteria (689 vs. 664) and 32 more in archaea (597 vs. 565).”**

Minor points:

3) The Methods do not mention what dilation sizes were used in the gene model CNN. d = 2^i ?

**Good catch. We have added this information to the text as well as figure 1.**

4) To train the start site model, negative training examples were taken to be the start site codons after the annotated start site of the positive training ORFs. Isn't that quite risky since start sites are notoriously hard to annotate and might be frequently wrong? Wouldn't it be better to use start codons within the negative ORF training examples?

**Ultimately, the start site predictor is used to choose between possible start sites within the same reading frame for a given gene, as all potential start sites must be in the same reading frame as the stop codon. We trained on downstream sites in the true reading frame because those data would be most similar to the actual decision made by the model during gene finding, where all potential start sites are in the positive ORF training example frame.**

**Even so, the start site data are quite noisy and the model never achieved nearly as low a loss as was observed with the gene model. Given the myriad of biologically inspired heuristics used by other methods to choose start sites, in addition to the fundamental lack of low-noise ground truth data, we felt it was best to leave start site prediction to later work. In truth, incorporating simpler start site models from other programs may be the best path forward, as the data may be too noisy and ground-truth too lacking for a complex model to show a significant improvement. We hope the last paragraph of the discussion will show this viewpoint.**


5) Whereas it is stressed in the abstract that Prodigal and Glimmer3 need to be pretrained on each genome to achieve optimal results, the Methods section does not mention if such pretraining was employed.

**Yes, it was. We have added this information to the methods:**

**“To benchmark gene finding performance, Glimmer3 and Prodigal were run with default settings and allowed to train on each genome in the test set.”**

6) Please explain why 'Efficiently training a temporal convolutional network requires sequences of the same 96 length.'

**We have removed this sentence from the manuscript.**

**This was intended more as a technical note, as passing tensors to the GPU for training requires the length of sequence to be the same in a single batch, and larger batches lead to speed improvements. However, we do not believe there is a mathematical reason training data must be the same size, and this note did not add much useful information.**

7) Why is Balrog so slow? I count 20 * 8 * 32 * 8 = 41920 parameters. Since predictions can be done in parallel on the GPU, that should take a few seconds, not minutes, for the few ten thousand translated ORFs longer than 60 codons. Could it be that each convolutional filter is not only computed once per input window of length k, as it should, but 100-k+1 times (where 100 is the length of the sequences used for training)?

**This is definitely true, the network is small enough that the number of parameters did not justify the long run time. Balrog was much slower than it needed to be for a few reasons which have been largely solved in the C++ version.**

**One of the largest issues was that each ORF was scored independently. Because long ORFs may have many possible start codons, which were treated as different ORFs, much of the same sequence was scored repeatedly. We have added a step to allow scoring only the longest ORF for each stop codon, then indexing into the predicted probability vector to recover predictions for all ORFs based on their start codon position.**

**This sped up scoring by the neural network significantly, to the point that most of the time spend by Balrog is now spent on various other necessary parts of the code, e.g. translation into amino acids, converting the sequence into one-hot tensors so they can be scored, etc. Many of these steps may still benefit from optimization, though we hope the new version will be acceptable for many users.**

**The current C++ version is moderately faster than the python version in wall-clock time even while running on a $200 cpu rather than a $3000 gpu.**

8) Line 13: Delete '32 ∗ L hidden units per layer, and'

**We have deleted this erroneous text.**

9) Please comment in the discussion on why you did not use a transformer architecture.

**Transformers are very interesting and in fact were used in our first attempt at a universal gene finder in 2019 (archived here** [**https://github.com/salzberg-lab/Smaug**](https://github.com/salzberg-lab/Smaug)**). Transformers seemed to work very well as well as a gene model, but we ultimately found the TCN achieved the same or better performance with significantly less overhead in time and memory. Transformers worked, but prokaryotic gene finding does not seem to require such a relatively complex architecture to achieve superior performance.**

**It is possible future work similar to Balrog may improve as transformer architectures become smaller and more effective, though our initial impressions were that more could be gained by integrating alternative sources of information (kmer matching, MMSeqs2 search) rather than a larger neural network. Note that we did not add this to the discussion because wanted to stay focused on the algorithm that we implemented.**
